# Supplementary material for: The interactome of intact mitochondria by cross-linking mass spectrometry provides evidence for coexisting respiratory supercomplexes
Source: Mol Cell Proteomics. 2017 Dec 8;17(2):216–32. doi: 10.1074/mcp.RA117.000470 (PMC5795388; doi:10.1074/mcp.RA117.000470)
Supplement: Supplemental Data [file supp_RA117.000470_133922_0_supp_23978_fzffwf.zip › spectra_annotation/mito_spectra_annotation/253-2-8-1-12-1.pdf]

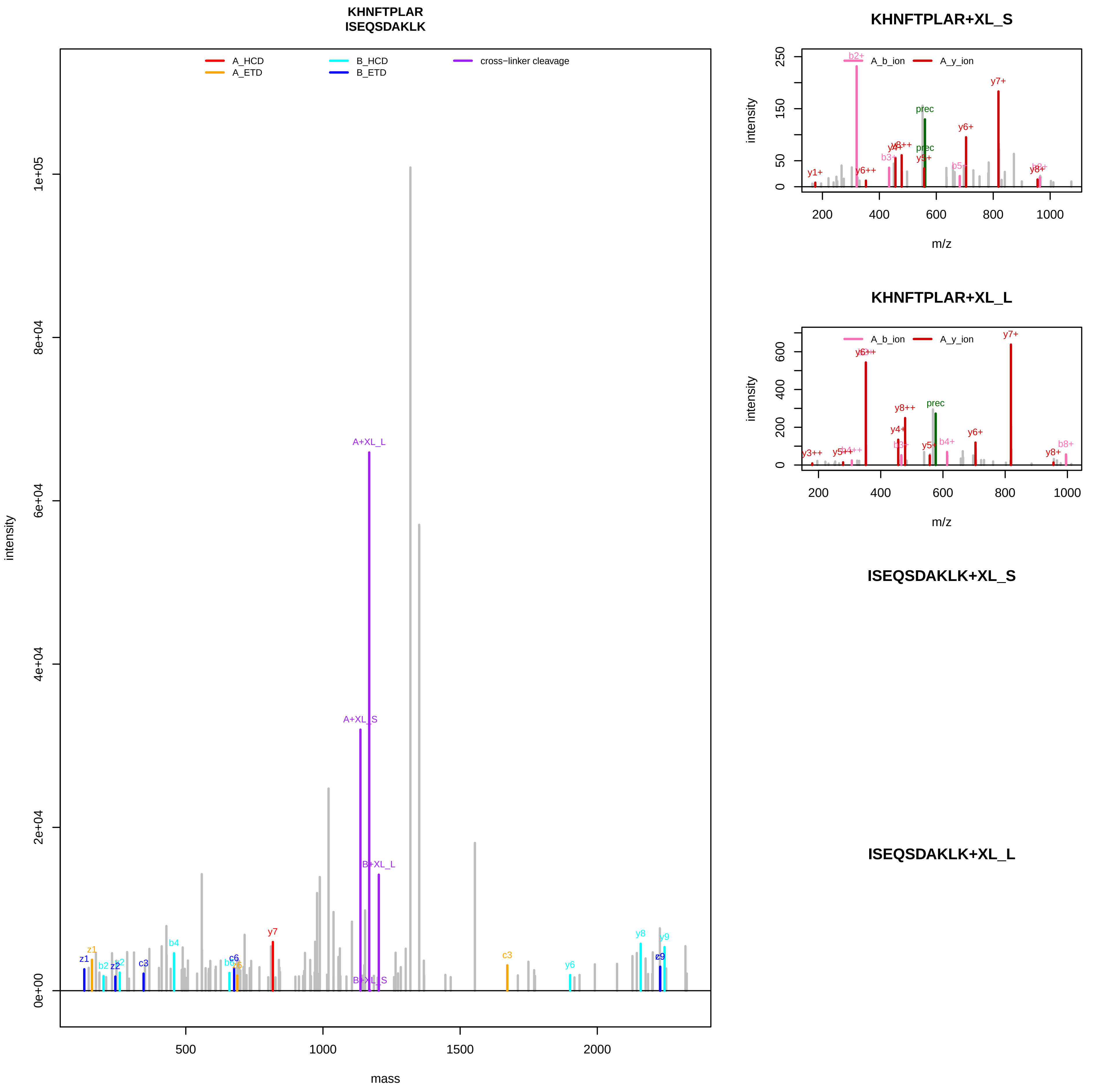

KHNFTPLAR
ISEQSDAKLK
A_HCD
A_ETD
B_HCD
B_ETD
cross−linker cleavage
intensity
mass
0e+00
2e+04
4e+04
6e+04
8e+04
1e+05
500
1000
1500
2000
A+XL_L
A+XL_S
B+XL_L
B+XL_S
z1
b2
z2
y2
c3
b4
b6
c6
y7
c3
y6
y8
y9
z9
KHNFTPLAR+XL_S
A_b_ion
A_y_ion
b2+
y7+
prec
y6+
y4+
y8++
b3+
y5+
y6++
y1+
b5+
y8+
b8+
intensity
m/z
KHNFTPLAR+XL_L
A_b_ion
A_y_ion
y7+
y6++
y8++
prec
y4+
b3+
y5+
b4+
y6+
y3++
y5++
b4++
y8+
b8+
intensity
m/z
ISEQSDAKLK+XL_S
ISEQSDAKLK+XL_L
